# Diabetes-Related Induction of the Heme Oxygenase System and Enhanced Colocalization of Heme Oxygenase 1 and 2 with Neuronal Nitric Oxide Synthase in Myenteric Neurons of Different Intestinal Segments

**DOI:** 10.1155/2017/1890512

**Published:** 2017-09-10

**Authors:** Lalitha Chandrakumar, Mária Bagyánszki, Zita Szalai, Diána Mezei, Nikolett Bódi

**Affiliations:** Department of Physiology, Anatomy and Neuroscience, Faculty of Science and Informatics, University of Szeged, Közép fasor 52, Szeged H-6726, Hungary

## Abstract

Increase in hyperglycaemia-induced oxidative stress and decreased effectiveness of endogenous defense mechanisms plays an essential role in the initiation of diabetes-related neuropathy. We demonstrated that nitrergic myenteric neurons display different susceptibilities to diabetic damage in different gut segments. Therefore, we aim to reveal the gut segment-specific differences in the expression of heme oxygenase (HO) isoforms and the colocalization of these antioxidants with neuronal nitric oxide synthase (nNOS) in myenteric neurons. After ten weeks, samples from the duodenum, ileum, and colon of control and streptozotocin-induced diabetic rats were processed for double-labelling fluorescent immunohistochemistry and ELISA. The number of both HO-immunoreactive and nNOS/HO-immunoreactive myenteric neurons was the lowest in the ileal and the highest in the colonic ganglia of controls; it increased the most extensively in the ileum and was also elevated in the colon of diabetics. Although the total number of nitrergic neurons decreased in all segments, the proportion of nNOS-immunoreactive neurons colocalizing with HOs was enhanced robustly in the ileum and colon of diabetics. We presume that those nitrergic neurons which do not colocalize with HOs are the most seriously affected by diabetic damage. Therefore, the regional induction of the HO system is strongly correlated with diabetes-related region-specific nitrergic neuropathy.

## 1. Introduction

There has been a dramatic increase in the incidence of various metabolic diseases, like hypertension, obesity, or diabetes, globally [1–3]. Diabetes mellitus has long been recognized as the major risk factor of cardiovascular diseases. Intensive glycemic control has reduced the risks of macro- and microvascular complications of diabetes; however, cardiovascular events remain the leading risk factor for mortality of diabetic patients worldwide [3, 4]. In addition to vascular complications, gastrointestinal (GI) symptoms such as gastroparesis, abdominal pain, diarrhoea, or constipation are also very common among diabetic patients. The relationship between these GI motility abnormalities and the presence of enteric neuropathy has been well documented in humans [5–7] as well as in rodent models [8, 9]. We have also investigated for years the involvement of those nitrergic myenteric neurons in these GI complications which have a key role in regulating gut motility. According to our previous results, the nitrergic neurons located in different gut segments display different susceptibilities to diabetic damage and different levels of responsiveness to insulin treatment [9]. These results highlight the importance of the neuronal microenvironment along the GI tract in the pathogenesis of diabetic nitrergic neuropathy. Working towards a better understanding of the molecular differences in the neuronal microenvironment, we have demonstrated that the mesenterial capillaries [10] supplying the myenteric ganglia and the faeces-associated microbiota [11] are also serious targets of diabetic injuries in the same rat model. Diabetes-related microvascular complications and alterations in the composition of gut microbiota are also region specific in the different intestinal segments and are closely correlated with region-dependent nitrergic myenteric neuropathy.

It is well known that hyperglycaemia is clearly the culprit in the pathogenesis of diabetic complications, and there are convincing evidences that the onset of diabetes and its symptoms are closely associated with oxidative stress [12, 13]. Moreover, hyperglycaemia increases the formation of advanced glycation end products and inflammatory events [14, 15]. There is also evidence that different subpopulations of myenteric neurons have selective responses to diabetic oxidative stress [16]. Besides an increased formation of reactive oxygen species (ROS), another main problem is the serious attenuation of antioxidant defense mechanisms. Among the endogenous antioxidants, the heme oxygenase (HO) system is recently emerging as an important player in diabetes and in GI inflammation [17–23]. HO is a microsomal enzyme with an essential role in heme catabolism producing biologically active carbon monoxide, biliverdin, and free iron [21, 24, 25]. Two main isoforms, HO1 and HO2, have been characterized as products of two different genes with distinct tissue- and cell-specific expression patterns [24, 26]. HO2 is considered to be a constitutive isozyme most highly expressed in neuronal tissues contributing to cell homeostasis, whereas HO1 is thought to be an inducible form with relatively low expression in most tissues [21, 24, 27]. Induction of the HO1 protein was shown to protect against a variety of stress conditions such as ischaemia [28], hypoxia [29], and ROS [30]. In addition, treatment with antioxidants minimizes or prevents the development of widespread complications in diabetic patients [31, 32]. The induction of HO1 decreased ROS, rapidly restored neuronal nitric oxide synthase (nNOS) expression, and reversed diabetic gastroparesis in mice [33]. It has also been reported that induction of HO1 reduces glomerular injury and apoptosis in diabetic spontaneously hypertensive rats [34]. In streptozotocin- (STZ-) induced diabetic rats, HO1 has been found to be induced in podocytes, protecting against apoptosis [35]. Moreover, in rat ileum, the antioxidant HO2 protects those NOS-containing neurons from oxidative stress in which it is colocalized [36]. Due to the beneficent effects of the HO system [23], these endogenous antioxidants can be the most important players in the prevention of oxidative injury and diabetic GI complications. However, HO induction may not always be beneficial: it can act also as a prooxidant mechanism depending on the disease milieu [37, 38], and the duration of HO stimulation may be a critical factor in triggering either the cytotoxic or the adaptive responses [39].

Based on earlier findings that as a result of the adequate oxidative environment in the proximal intestine, inducing a deep anaerobic state in the distal intestine [40, 41], the colon was more susceptible to damage by oxidative stress [42]; we supposed that the intestinal region-dependent colocalization of these endogenous antioxidants with nitrergic myenteric neurons results in different degrees of protection against chronic hyperglycaemia-induced oxidative stress. Therefore, our primary aim was to determine the gut segment-specific differences in the expression of the two HO isoforms, HO1 and HO2, and we also planned to evaluate the colocalization of these antioxidants with nNOS in myenteric neurons of different intestinal segments of chronic diabetic and control rats. To reflect the immediate alterations after the onset of hyperglycaemia, it was also our goal to study the serum level of HOs in some early stages of hyperglycaemia.

## 2. Materials and Methods

### 2.1. Animal Models

In all procedures involving experimental animals, the principles of laboratory animal care (NIH Publication No. 85–23, revised 1985) were strictly followed, and all the experiments were approved in advance by the Local Ethics Committee for Animal Research Studies at the University of Szeged. Adult male Wistar rats (Crl: WI BR; Toxi-Coop Zrt.), kept on standard laboratory chow (Farmer-Mix Kft., Zsámbék) and with free access to drinking water, were used throughout the experiments.

### 2.2. Acute Diabetic Rat Model

For the acute hyperglycaemic experiments, the rats (250–350 g) were divided randomly into four groups: STZ-induced 3-day diabetics (*n* = 5), 5-day diabetics (*n* = 5), 10-day diabetics (*n* = 5), and sex- and age-matched controls (*n* = 5). Hyperglycaemia was induced as described previously [9, 10]. The animals were considered diabetic if the nonfasting blood glucose concentration was higher than 18 mM. The blood glucose level and weight of each animal were measured daily.

### 2.3. Chronic Diabetic Rat Model

For the chronic hyperglycaemic experiments, the rats (210–260 g) were divided randomly into two groups: STZ-induced 10-week diabetics (*n* = 6) and sex- and age-matched controls (*n* = 5). Hyperglycaemia was induced as described previously [9, 10]. The animals were considered diabetic if the nonfasting blood glucose concentration was higher than 18 mM. The blood glucose level and weight of each animal were measured weekly during the 10-week experimental period.

### 2.4. Blood Collection and Measurement of Serum HO1 and HO2 Concentrations of Acute and Chronic Diabetic Animals

The blood samples were collected from the tail vein of each animal (BD Vacutainer SST II Advance) after 3 days, 5 days, and 10 days as well as 10 weeks of STZ treatment. Then, they were centrifuged at 3200 rpm for 10 min at room temperature, and the supernatants were stored in aliquots at −80°C until use. The HO1 and HO2 levels of the serum samples of acute and chronic diabetic animals were determined by means of quantitative ELISA according to the manufacturer's instructions (SunRed Biotechnology, Shanghai, China). Optical density was measured at 450 nm (Benchmark Microplate Reader; Bio-Rad, Budapest, Hungary). The serum HO1 and HO2 concentrations were expressed as ng/ml.

### 2.5. Tissue Handling

Ten weeks after the onset of hyperglycaemia, the animals of the chronic diabetic experiment were killed by cervical dislocation under chloral hydrate anaesthesia (375 mg/kg ip). The gut segments of the control and STZ-induced diabetic rats were dissected and rinsed in 0.05 M phosphate buffer (PB; pH 7.4). Samples were taken from the duodenum (1 cm distal to the pylorus), the ileum (1 cm proximal to the ileocaecal junction), and the proximal colon and processed for immunohistochemical studies and enzyme-linked immunosorbent assays (ELISA). For double-labelling immunohistochemistry, the intestinal segments were cut along the mesentery, pinched flat, and fixed overnight at 4°C in 4% paraformaldehyde solution buffered with 0.1 M PB (pH 7.4). The samples were then washed, the mucosa, submucosa, and circular smooth muscle were removed, and whole-mounts with the myenteric plexus adhering to the longitudinal muscle were prepared. For the ELISA, the gut segments were cut along the mesentery and pinched flat. The layer of mucosa and submucosa was removed, and the residual material was snap frozen in liquid nitrogen and stored at −80°C until use.

### 2.6. Double-Labelling Fluorescent Immunohistochemistry

For double-labelling immunohistochemistry, whole-mount preparations derived from different gut segments were immunostained with nNOS and HO1 or HO2. Briefly, after blocking of the preparations in PB containing 0.1% bovine serum albumin, 10% normal goat serum, and 0.3% Triton X-100, they were incubated overnight with anti-nNOS (rabbit; Cayman Chemical, Ann Arbor, MI, USA; final dilution 1 : 200) and anti-HO1 (mouse; Novus Biologicals Europe, Abington, UK; final dilution 1 : 200) or anti-HO2 (mouse; Santa Cruz Biotechnology, Inc., Dallas, Texas, USA; final dilution 1 : 50) primary antibodies. After washing in PB, whole-mounts were incubated with antirabbit Alexa Fluor 488 (Life Technologies Corporation, Molecular Probes, Inc., Eugene, OR; final dilution 1 : 200) and antimouse Cy™3 (Jackson ImmunoResearch Laboratories Inc., Baltimore Pike, PA; final dilution 1 : 200) secondary antibodies for 4 hours. All incubations were carried out at room temperature. Negative controls were performed by omitting the primary antibody, when no immunoreactivity was observed. Whole-mounts were mounted on slides in EverBrite™ Mounting Medium (Biotium, Inc., Hayward, CA), observed and photographed with an Olympus BX51 fluorescent microscope equipped with an Olympus DP70 camera. Fifty myenteric ganglia were taken from each intestinal segment from each experimental group, and the numbers of nNOS-IR, HO1- or HO2-IR neurons, and those myenteric neurons in which the two markers were colocalized (per ganglia) were counted. The percentage of ganglia containing HO-IR or nNOS-HO-IR neurons or none of these was also determined.

### 2.7. Measurement of Tissue HO1 and HO2 Concentrations

The intestinal tissue samples were frozen in liquid nitrogen, crushed into powder in a mortar, and homogenized in 500 *μ*l homogenizing buffer (100 *μ*l Protease Inhibitor Cocktail [Sigma-Aldrich, St. Louis, MO] in 20 ml 0.05 M PB). Tissue homogenates were centrifuged at 5000 rpm for 20 min at 4°C. The HO1 and HO2 levels of the intestinal tissue samples were determined by means of quantitative ELISA according to the manufacturer's instructions (SunRed Biotechnology, Shanghai, China). Optical density was measured at 450 nm (Benchmark Microplate Reader; Bio-Rad, Budapest, Hungary). The tissue HO1 and HO2 concentrations were expressed as ng/mg protein.

### 2.8. Bradford Protein Micromethod for the Determination of Tissue Protein Content

For the determination of protein content of tissue samples, a commercial protein assay kit was used. Bradford reagent was added to each sample. After mixing and following 10 min incubation, the samples were assayed spectrophotometrically at 595 nm. Protein level was expressed as mg protein/ml.

### 2.9. Statistical Analysis

Statistical analysis was performed with one-way ANOVA and the Newman-Keuls test. All analyses were carried out with GraphPad Prism 6.0 (GraphPad Software, La Jolla, CA, USA). A probability of *P* < 0.05 was set as the level of significance. All data were expressed as means ± SEM.

## 3. Results

### 3.1. Disease Characteristics in Diabetic Rats

The general characteristics of the control and STZ-treated animals from the acute and chronic diabetic experiments 3 days, 5 days, 10 days, and ten weeks after the STZ treatment are shown in Tables 1 and 2. The chronic diabetic rats were characterized by a significantly reduced body weight and an increased blood glucose concentration (23.31 ± 0.53 mM) as compared to the age- and sex-matched controls.

### 3.2. Serum Level of HOs in Acute and Chronic Diabetic Animals

**Figure 1:**
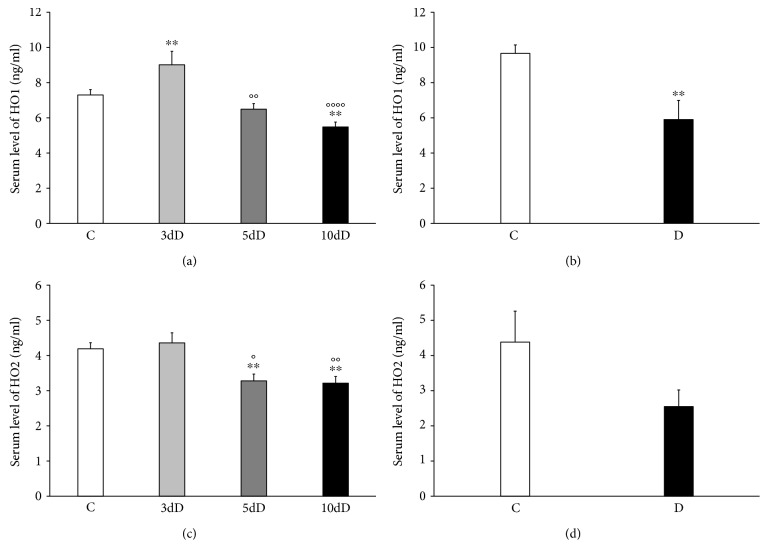
The serum level of HO1 and HO2 in acute (a, c) and chronic (b, d) diabetic rats. The HO1 expression increased significantly in the 3-day diabetics with a recovery in the 5-day-diabetic rats, and it decreased further in the 10-day-diabetic animals as compared to the controls (a). The serum HO1 level was almost the same in the 10-week and the 10-day diabetics (a, b). The serum concentration of HO2 was also decreased in 5-day-, 10-day-, and 10-week-diabetic animals as compared to their controls (c, d). Data are expressed as means ± SEM. ^∗∗^*P* < 0.01 (compared to the controls), ^o^*P* < 0.05; ^oo^*P* < 0.01; ^oooo^*P* < 0.0001 (compared to the 3-day diabetics). C: controls; 3dD: 3-day diabetics; 5dD: 5-day diabetics; 10dD: 10-day diabetics; D: 10-week diabetics.

In the serum samples, the HO1 expressed at higher level than the HO2 (7.3 ± 0.32 versus 4.19 ± 0.19 ng/ml) in the healthy animals of acute study (Figures 1(a) and 1(c)). Three days after the STZ treatment, the HO1 expression increased significantly (*P* < 0.01) as compared to controls, following a recovery in the 5-day-diabetic rats. Moreover, it decreased further in the 10-day diabetics as compared to control animals (Figure 1(a)). The expression of HO1 was also decreased in the diabetic rats of the chronic study as compared to controls; furthermore, the serum HO1 concentration was not significantly different in 10-week and 10-day diabetics (5.9 ± 1.09 versus 5.48 ± 0.3 ng/ml; Figures 1(a) and 1(b)).

In the case of HO2, similar changes were detected in the serum samples of acute and chronic study. Briefly, the HO2 level was significantly decreased in 5-day-, 10-day-, and 10-week-diabetic animals as compared to their controls (Figures 1(c) and 1(d)).

### 3.3. Evaluation of Myenteric Ganglia Representing HO-IR and nNOS-HO-IR Neurons

**Figure 2:**
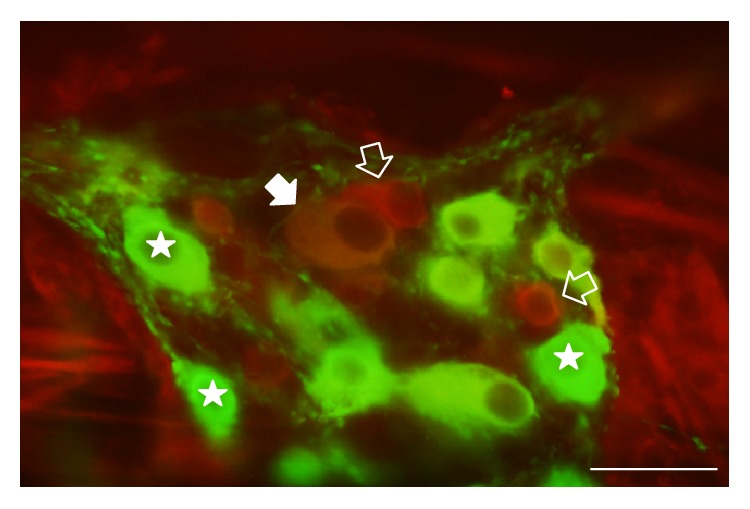
Representative fluorescent micrograph of a whole-mount preparation of myenteric ganglia from the colon of a diabetic rat after nNOS-HO1 immunohistochemistry. Stars indicate neurons that labelled for nNOS only, open arrows show neurons that labelled for HO1 only, and the solid arrow points to a myenteric neuron that double-labelled for both nNOS and HO1. Scale bar: 50 *μ*m.

**Figure 3:**
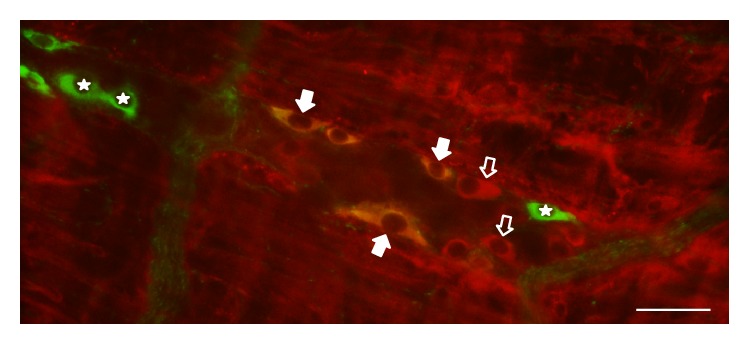
Representative fluorescent micrograph of a whole-mount preparation of myenteric ganglia from the duodenum of a diabetic rat after nNOS-HO2 immunohistochemistry. Stars indicate neurons that labelled for nNOS only, open arrows show neurons that labelled for HO2 only, and solid arrows point to myenteric neurons that double-labelled for both nNOS and HO2. Scale bar: 50 *μ*m.

Nitrergic myenteric neurons were labelled by nNOS immunohistochemistry, whereas HO-representing neurons were visualized by HO1 and HO2 immunohistochemistry. The densities of HO1- and HO2-IR- as well as HO1- and HO2-representing nitrergic neurons were evaluated per ganglia of the duodenum, ileum, and colon in control and diabetic rats (Figures 2 and 3).

**Figure 4:**
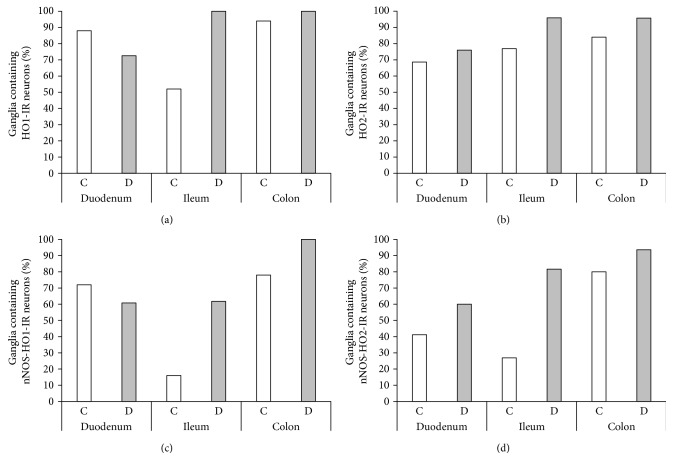
Percentage of myenteric ganglia containing HO1-IR (a), HO2-IR (b), nNOS-HO1-IR (c), and nNOS-HO2-IR (d) neurons. Only half of the ileal ganglia contained HO1-IR neurons, and from these only 16% of the ganglia represented double-stained neurons in the control rats. In the diabetic group, all of the ileal and colonic ganglia included HO1-IR neurons, and from these more than 60% of ileal and 100% of colonic ganglia also represented the double-stained neurons. More than 70% of ganglia represented HO2-IR neurons under both experimental conditions, and a large increase in the proportion of ganglia containing double-stained neurons was revealed in diabetic state. C: controls; D: diabetics.

The proportion of HO1-including myenteric ganglia was different in the various intestinal segments in control and diabetic rats. They were observed in the highest percentage in the colon (94%) and in the duodenum (88%) of controls. However, only half of the ileal ganglia contained HO1-IR neurons and from these ganglia only 16% represented nNOS-HO1 colocalized neurons in the control rats (Figures 4(a) and 4(c)).

In the diabetic group, all of the ileal ganglia included HO1-IR neurons, and more than 60% of them also represented double-stained neurons. In the diabetic colon, all of the myenteric ganglia included HO1-IR and also double-stained neurons (100% versus 78% in controls), whereas in the diabetic duodenum, the percentage of ganglia containing HO1- and nNOS-HO1-IR neurons was slightly decreased (88% versus 73% and 72% versus 61% in controls, resp.) (Figures 4(a) and 4(c)).

The proportion of ganglia representing HO2- and nNOS-HO2-IR neurons also varied in a region-dependent way. In controls, 70–80% of the ganglia included HO2-IR neurons in all gut segments with increased rate in diabetic animals (Figure 4(b)). The lowest proportion of nNOS-HO2-representing ganglia was observed in the ileum (27%) and the highest in the colon (80%) of the control rats. In the diabetic group, the rate of nNOS-HO2-including ganglia increased in all intestinal segments; however, the greatest, 3-fold increase was observed in the ileum (Figure 4(d)).

### 3.4. Total Number of HO1-IR- and HO1-Representing Nitrergic Neurons

**Figure 5:**
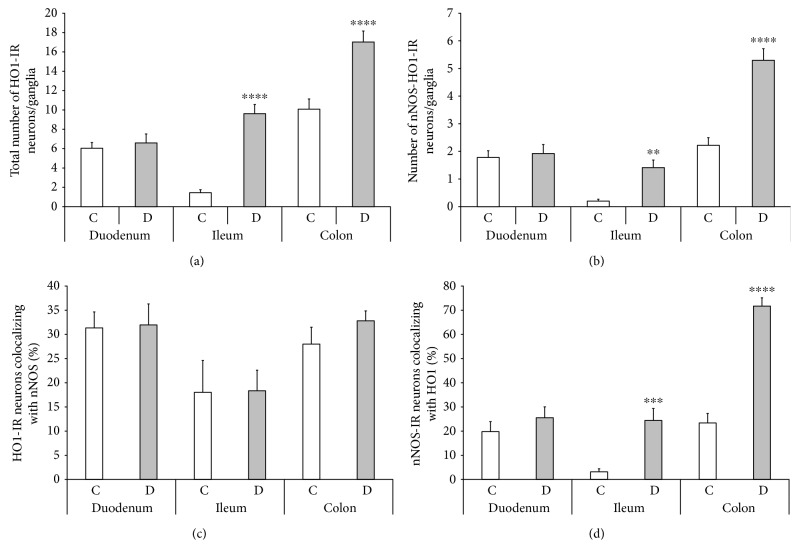
Quantitative evaluation of the total number of HO1-IR neurons (a), the number of nNOS-HO1-IR neurons (b), the proportion of HO1-IR neurons colocalizing with nNOS (c), and the proportion of nNOS-IR neurons colocalizing with HO1 (d) in the myenteric ganglia of the duodenum, ileum, and colon of control and diabetic rats. Except for the duodenum, the total number of HO1-IR neurons and the number of nNOS-HO1-IR myenteric neurons displayed a robust increase in the ileum and colon of diabetic rats. The percentage of HO1-IR neurons colocalizing with nNOS did not change in the different intestinal segments. However, the proportion of nNOS-IR neurons colocalizing with HO1 exhibited a nearly 8-fold increase in the ileum and a more than 2-fold increase in the colon of diabetics. Data are expressed as means ± SEM. ^∗∗^*P* < 0.01, ^∗∗∗^*P* < 0.001, and ^∗∗∗∗^*P* < 0.0001 (between controls and diabetics). C: controls; D: diabetics.

The presence of HO1-IR neurons varied distinctly in the different gut segments even in the control animals. The least HO1-IR cells were counted in the ileal ganglia (1.44 ± 0.31), whereas 4-fold more HO1-IR neurons were observed in the duodenum (6.04 ± 0.59) and 7-fold more in the myenteric ganglia of the colon (10.08 ± 1.05). Except for the duodenum, the total number of HO1-IR neurons displayed a robust increase in the ileum (*P* < 0.0001) and colon (*P* < 0.0001) of diabetic rats as compared to the control data. The largest increase was observed in the ileum, where almost 7-fold more HO1-IR neurons were counted in the diabetics relative to controls (9.62 ± 0.95 versus 1.44 ± 0.31) (Figure 5(a)).

The number of nNOS-HO1-IR myenteric neurons showed a very similar distribution in the control group with the lowest value in the ileal and the highest in the colonic ganglia. Moreover, the number of nNOS-HO1 colocalized neurons was also elevated 7-fold in the ileum of diabetics as compared to controls (1.41 ± 0.27 versus 0.2 ± 0.07). In the diabetic colon, the number of nNOS-HO1 colocalized neurons was more than doubled, whereas in the duodenum of diabetic rats it remained unchanged (Figure 5(b)). Since the number of total HO1-IR and also that of the nNOS-HO1-IR neurons represented similar alterations in the different gut segments of control and diabetic rats, therefore the proportion of HO1-IR neurons colocalizing with nNOS did not change in any of the different intestinal regions. In the ileum, only 18% of HO1-IR neurons were colocalized with nNOS, whereas this ratio was around 30% in the ganglia of the duodenum and colon under both experimental conditions (Figure 5(c)).

Although the total number of nNOS-IR neurons decreased in all intestinal segments (9 ± 0.39 versus 7.53 ± 0.29 in the duodenum, *P* < 0.01; 6.32 ± 0.35 versus 5.78 ± 0.27 in the ileum; 9.5 ± 0.48 versus 7.38 ± 0.42 in the colon, *P* < 0.001), the proportion of nNOS-IR neurons colocalizing with HO1 exhibited a nearly 8-fold increase in the ileum and a 3-fold increase in the colon of diabetics. This ratio was barely 3% in the ileum and 23% in the colon of the control rats, whereas it was 24% and 72% in the diabetics, respectively (Figure 5(d)).

### 3.5. Total Number of HO2-IR- and HO2-Representing Nitrergic Neurons

**Figure 6:**
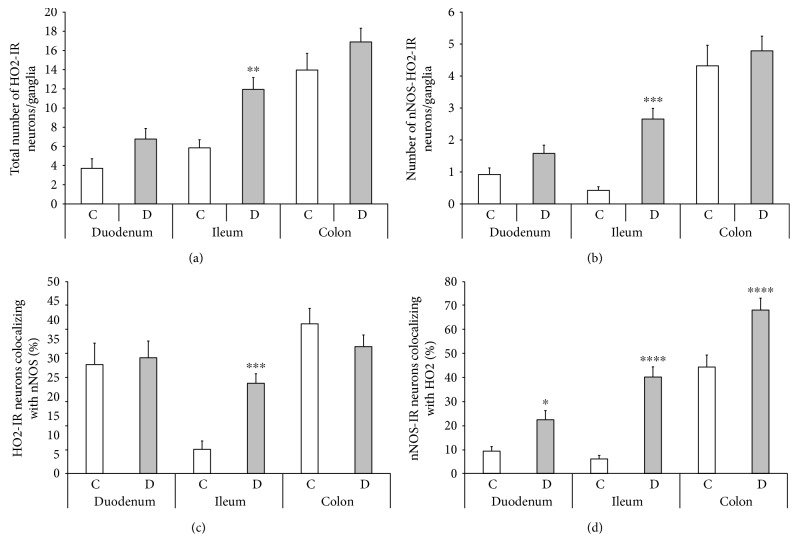
Quantitative evaluation of the total number of HO2-IR neurons (a), the number of nNOS-HO2-IR neurons (b), the proportion of HO2-IR neurons colocalizing with nNOS (c), and the proportion of nNOS-IR neurons colocalizing with HO2 (d) in the myenteric ganglia of the duodenum, ileum, and colon of control and diabetic rats. The total number of HO2-IR neurons and the number of nNOS-HO2-IR myenteric neurons largely increased in the ileum of diabetic rats. The percentage of HO2-IR neurons colocalizing with nNOS was elevated only in the diabetic ileum. However, the proportion of nNOS-IR neurons colocalizing with HO2 was enhanced in each gut segment of diabetics. The largest increase, more than 6-fold, was observed in the ileal ganglia. Data are expressed as means ± SEM. ^∗^*P* < 0.05, ^∗∗^*P* < 0.01, ^∗∗∗^*P* < 0.001, and ^∗∗∗∗^*P* < 0.0001 (between controls and diabetics). C: controls; D: diabetics.

The distribution of HO2-IR neurons also displayed a region-dependent pattern in the control rats. The bulk of the HO2-IR neurons was located in the colonic ganglia (13.96 ± 1.73), whereas less than half as many were counted in the myenteric ganglia of the ileum and the duodenum. In the diabetic rats, the total number of HO2-IR neurons increased in the different gut segments; however, the increase was significant only in the ileum (*P* < 0.01), where the HO2-IR neuronal number was doubled relative to controls (11.94 ± 1.24 versus 5.85 ± 0.85) (Figure 6(a)). The number of nNOS-HO2-IR myenteric neurons represented similar expression in controls, where the majority of the colocalized neurons also occurred in the ganglia of the colon (4.32 ± 0.64), whereas an average of 0-1 cells was found in the ileal and duodenal ganglia. Moreover, the number of nNOS-HO2 colocalized neurons was also elevated more than 6-fold in the ileum of diabetics as compared to controls (2.65 ± 0.34 versus 0.42 ± 0.11; *P* < 0.001) (Figure 6(b)). Since the number of total HO2-IR and also that of the nNOS-HO2-IR neurons did not alter significantly in the duodenum and colon of control and diabetic rats, therefore the proportion of HO2-IR neurons colocalizing with nNOS remained unchanged in these intestinal regions. In the duodenum 28–30%, whereas in the colon 33–39% of HO2-IR neurons were colocalized with nNOS under both experimental conditions. However, this proportion was 6% in the ileum of the control rats with a pronounced increase in the same segment of diabetics (24%; *P* < 0.001) (Figure 6(c)).

Although the total number of nNOS-IR neurons decreased in all intestinal regions (10.39 ± 0.51 versus 7.96 ± 0.44 in the duodenum, *P* < 0.01; 7.33 ± 0.42 versus 6.23 ± 0.22 in the ileum, *P* < 0.05; 10.48 ± 0.69 versus 8.08 ± 0.69 in the colon, *P* < 0.01), however, the proportion of nNOS-IR neurons colocalizing with HO2 was definitely increased in all gut segments of the diabetic group. The largest, nearly a 7-fold increase, in this ratio was observed in the ileum (40% versus 6%), but significant increases were also shown in the duodenum (22% versus 9%) and the colon (68% versus 44%) of diabetics as compared to controls (Figure 6(d)).

### 3.6. Tissue Level of HOs

**Figure 7:**
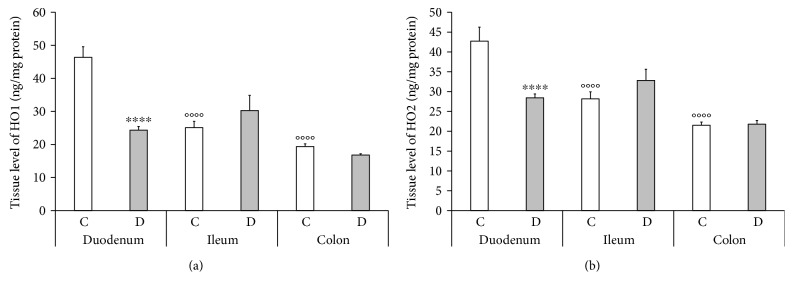
The tissue level of HO1 (a) and HO2 (b) in the different gut segments of control and diabetic rats. The levels of HO1 and HO2 were the highest in the tissue homogenates from the duodenum of controls and decreased to the distal part of the GI tract. In the diabetic rats, a statistically intense decrease was observed in the level of HO1 and HO2 in the tissue homogenates of the duodenum but not in those of the ileum and colon. Data are expressed as means ± SEM. ^∗∗∗∗^*P* < 0.0001 (between controls and diabetics), ^oooo^*P* < 0.0001 (between different gut segments of controls). C: controls; D: diabetics.

In the tissue homogenates including the smooth muscle layers of the intestinal wall and the myenteric plexus, the expressional patterns of HO1 and HO2 were strictly region dependent in the healthy animals of chronic study. The highest concentrations of HO enzymes were detected in the tissue homogenates of the control duodenum with 46.35 ± 3.22 ng/mg protein in the case of HO1 and 42.72 ± 3.53 ng/mg protein in the case of HO2 (Figures 7(a) and 7(b)). In the ileum and colon of controls, the tissue level of HOs was significantly lower than in the duodenum. In the 10-week diabetics, the duodenum was the only gut segment where the tissue levels of HOs decreased strongly (24.35 ± 1.12 ng/mg protein in the case of HO1 and 28.44 ± 0.97 ng/mg protein in the case of HO2), whereas they remained unchanged in the ileum and colon (Figures 7(a) and 7(b)).

Since the changes in the total number of HO-IR- and HO-representing nitrergic neurons was the most elevated in the ileum of 10-week-diabetic rats, therefore we chose this particular gut segment also from the acute study to measure the tissue level of HOs. In the tissue homogenates of the ileum, the HO2 expressed at higher level than the HO1 (26.94 ± 0.81 versus 20.55 ± 1.49 ng/mg protein) in the healthy animals of the acute study. The acute hyperglycaemia did influence neither the HO1 nor the HO2 expression 3, 5, or 10 days after the STZ treatment as compared to controls (19.35 ± 0.98 versus 18.09 ± 0.77 versus 18.76 ± 1.23 ng/mg protein in the case of HO1 and 27.76 ± 0.92 versus 28.66 ± 1.02 versus 27.72 ± 1.31 ng/mg protein in the case of HO2, resp.).

## 4. Discussion

Based on our findings of immediate alterations after the onset of hyperglycaemia, we presume that the increased HO1 level in the serum of 3-day-diabetic rats can be a result of the stress response caused by STZ treatment. This acute HO response does not appear in the intestinal tissue homogenates (at least in the ileum), which further strengthens our presumption. This acute stress response appeared firstly in the systemic circulation and not in the tissue of the gut and then it declines gradually here in the 5- and 10-day-diabetic rats. Moreover, the serum concentration of HO1 and HO2 were almost the same in 10-day- and 10-week-diabetic animals.

In support of our earlier finding that the susceptibility of nitrergic myenteric neurons to experimentally induced diabetes is strictly regional [9], the present study provides evidence of gut segment-specific diabetes-related induction of the endogenous HO system and also the intestinal region-dependent enhanced colocalization of HO1 and HO2 with nNOS in myenteric neurons.

The occurrence of HO1- or HO2-containing myenteric ganglia, furthermore the presence of those ganglia which include nNOS-HO1 or nNOS-HO2 colocalized neurons, was the most pronounced in the colon and the slightest in the ileum of the control rats. Similarly, the number of HO1- or HO2-IR and nNOS-HO1 or nNOS-HO2-IR neurons was the highest in the colon and the lowest in the ileum under control conditions. Interestingly, in the duodenum of controls, although the number of HO1-IR or HO2-IR and nNOS-HO1-IR or nNOS-HO2-IR neurons was less than in the colon, still the presence of either HO-IR or nNOS-HO-IR neurons representing myenteric ganglia was nearly as explicit as in the colon.

Even under physiological conditions, these gut segment-specific differences raise the question of why the myenteric ganglia and nitrergic neurons are distributed differently, depending on their intestinal location. It is well established that the anatomical, functional, and pathological regionality of the GI tract develops under strict genetic control [43, 44], which in itself can be responsible for the unique features of the enteric neurons in a healthy state and for their different susceptibilities to pathological insults in different gut segments. Emerging evidences [45, 46] confirm that the different degrees of susceptibility of enteric neurons to a pathological stimulus such as hyperglycaemia might be related to the prevalence of bacteria in the different parts of the GI tract, which among others give rise to major differences in the intestinal redox status and the oxygen supply of the small and large intestine [47–49]. The microflora of facultative bacteria expands during infancy and creates a reducing environment that supports the population of the gut by anaerobic strains, inducing a deep anaerobic state in the distal intestine. The prooxidant environment of the colon as compared to the small intestine may also contribute to greater cancer susceptibility [48]. In our recent study [13], we already demonstrated evidence of gut region-specific accumulation of reactive oxygen species and also that enhanced oxidative stress leads to regionally distinct activation of antioxidant and apoptotic marker molecules in proximal and distal part of the gut in rats with STZ-induced diabetes. There was no significant change in peroxynitrite level in the duodenum in any of the examined groups. However, in the diabetic colon, the peroxynitrite level was significantly increased, and the presence of severe necrosis was also confirmed by electron microscopy [13]. These data also suggest that the distal part of the gut is more vulnerable than the proximal to oxidative stress.

Therefore, we suppose that in the colon, where the baseline oxidative environment is far from optimal, the basal expression of HOs as essential players of the endogenous defense mechanisms is the most pronounced. In another study by Battish et al. [50], region-specific coexistence of HO2 with nNOS was also observed in the opossum anorectum, where the high percentage of colocalization in the myenteric plexus fell from almost 100% in the internal anal sphincter to 56% proximally in the rectum. This supports our findings that 44% of nNOS-IR neurons are also colocalized with HO2 in the colon of the control rats.

We also suggest that in the ileum the presence of the HO proteins is extremely low in controls, which may result in much lower protection against different pathological stimuli. Other groups have also demonstrated a hardly detectable expression of HO1 protein in mucosal epithelial cells of the ileum, and these were much more vulnerable to injuries by haemorrhagic shock [51] or septic damage [52]. Miller et al. [53] detected little if any immunoreactivity of HO1 in the human antrum and jejunum. However, Donat et al. [54] reported that only 10% of HO2-positive neurons contained NOS in rat ileum, which is in good correlation with our results.

We also assume that as a result of the adequate oxidative environment and definite baseline expression of HOs and their colocalization with nNOS in the myenteric ganglia in the proximal small intestine, the nitrergic neurons get greater protection and can tolerate hyperglycaemia-related oxidative stress better in the duodenum. This is supported by evidence that despite the impairment of nNOS pathways in STZ-induced diabetic rats, the nitrergic myenteric neurons did not die in the duodenum, unlike the other gut segments [9].

Moreover, the highest level of HO1 and HO2 expression in the tissue homogenates of the control duodenum (including the smooth muscle layers of the gut wall and the myenteric plexus between them) also highlights an intensified protective environment in this particular gut segment and emphasizes the importance of the neuronal microenvironment [10, 12]. Microsomal HO activity was also highest in the duodenal mucosa, where absorption of hemoglobin iron is reported to be most effective, and progressively fell in more caudal intestinal segments [55].

In diabetics, the number of HO1- or HO2-representing myenteric ganglia, as well as the number of those ganglia which contain nNOS-HO1 or nNOS-HO2 colocalized neurons, was markedly elevated in the ileum. Likewise, the number of HO1- or HO2-IR and nNOS-HO1 or nNOS-HO2-IR neurons was altered in a region-dependent manner in diabetic rats. The most intensified enhancement was also observed in the ileum of diabetics, which highlights the highest concern of this intestinal region in diabetes-related damage, also predicted in our earlier study [11]. Using a type 1 diabetic rat model to analyse the composition of faeces-associated microbiota, we demonstrated that only ileal samples from diabetic rats exhibited striking, but massive invasion (31%) of the pathogen genus Klebsiella [11]. It was recently reported that intestinal HO1 is induced by the enteric microbiota and modulates the macrophages bactericidal activity, suggesting its importance in maintaining homeostasis [56]. On the base of this, we suggest that the diabetes-related massive changes in the composition of the ileal gut microbiota [11] may contribute to the enhanced mucosal immune response and the greatest induction of endogenous HO system in this particular gut segment.

As regards the details of segment-specific diabetic alterations, in the diabetic ileum, both the HO1-IR and the nNOS-HO1-IR neuronal number was enhanced 7-fold, and the number of nNOS-HO2-IR neurons also increased 6-fold as compared to controls. Nevertheless, the total number of nNOS neurons decreased in all intestinal segments of diabetics, confirming our earlier findings [9]. Accordingly, much more nitrergic neurons also become HO-IR; thus, many of them start to product HO enzymes. Moreover, this also means that those nNOS-positive neurons which are not colocalized with HOs will be destroyed. Similar alterations were seen in the colon, where besides a 22% decrease in the nNOS number, a more than 50% increase was demonstrated in the number of nNOS-HO1-IR neurons, from which we assume that HO-containing nitrergic neurons enjoy higher protection, whereas the others are heavily affected by diabetic damage. In the diabetic duodenum, besides a decreasing number of nNOS neurons, the number of colocalized myenteric neurons did not alter significantly, leading to the same conclusion. However, based on the study of Izbéki et al. [9], in which the duodenum was the only gut segment where the decrease of NADPHd-positive neuronal number was not followed by a decrease in the number of total myenteric neurons, we suppose that in the present study, the reduced number of nNOS neurons means cell loss in the diabetic ileum and colon but a change in neurochemical character in the duodenum. Among others, the neuronal microenvironment-like capillaries of the gut wall or intestinal microbiota of the diabetic duodenum were broadly unharmed [10, 11].

Diabetic gastroparesis is a widely researched complication of diabetes, in which the interstitial cells of Cajal responsible for normal gastric emptying are largely damaged [7]. In the diabetic gastric fundus, the number of nNOS-IR neurons was decreased, nNOS expression was downregulated [57], and an impairment in nitrergic relaxation was also reported contributing to the development of diabetic gastroparesis in rats [58]. In mouse models of diabetes, increased expression of antioxidants such as HO1 protected interstitial cells of Cajal from oxidative stress and reversed diabetic gastroparesis [33]. Recent discovery shows that HO gives neuroprotection by controlling redox formation and reducing production of tumor necrosis factor alpha (TNF*α*) [59]. In the light of these findings, the role of the endogenous HO system in the protection of intestinal motility in diabetics is further strengthened. However, the literary data suggest that TNF may also be capable of exerting opposite effects, which could depend on parameters such as the site, degree, and duration of the ischemic period, the amount of TNF production, the expression level of the two TNF receptors, and the cellular environment of affected neurons [60]. It has been demonstrated that the involvement of the TNF/TNF-receptor system and opposing actions of the two TNF receptors resulting neuronal damage or promoting neuroprotection via different signalling pathways [60–62].

At present, only limited data are available about the diabetes-related expressional changes of HOs in different gut segments. In the STZ rat model, the nitrergic myenteric neurons that also express HO2 were more resistant to the effects of diabetes and less likely to undergo apoptosis [36]. In the diabetic ileum, this study revealed an increased size of neuronal cell body in nNOS-IR neurons, while HO2-IR neurons remained unaffected. Double-labelling studies confirmed that the diabetes-induced change in size was confined to nNOS-IR neurons that did not contain HO2.

The induction of the HO system by agents (such as several redox signals, hypoxia, or endotoxin) has been suggested to be an initial event in the development of an adaptive response to oxidative stress and inflammatory events in type 1 and type 2 diabetes [19, 63–66]. Bacterial endotoxin induces HO mRNA accumulation [67] and directly stimulates HO activity in macrophages and the liver [68]. Emerging evidence indicates that the upregulation of the HO system and related products increases pancreatic beta cell insulin release and reduces hyperglycaemia in different animal models [22]. The HO system is also upregulated in short term diabetes, leading to HO and NO interactions, which may modulate vascular function in the retina [69, 70]. Cheng et al. [71] reported that hypoxia-inducible factor-1*α* target genes like HO also contribute to retinal neuroprotection. It has also been demonstrated that NO and NO donors are capable of inducing HO1 protein expression, in a mechanism depending on the de novo synthesis of RNA and protein. Thus, it is postulated that NO may serve as a signaling molecule in the modulation of the tissue stress response [72].

## 5. Conclusions

In conclusion, our present findings emphasize that the endogenous HO system of the GI tract is also a target of diabetic damage. In addition, the evidences published here have revealed for the first time the intestinal region-dependent induction of the HO system as well as the segment-specific enhanced colocalization of HO1 and HO2 with nNOS in myenteric neurons. Obviously, the functional consequences of these diabetes-related alterations, namely, the possible interactions between the carbon monoxide and nitric oxide pathways produced by them in enteric neurons to regulate various GI functions demand further investigations.

## Figures and Tables

**Table 1:** Weight and glycaemic characteristics of the animal groups of the acute diabetic experiment.

	Body weight (g) ± SEM		Blood glucose concentration (mM) ± SEM	
	Initial	Final	Initial	Final (average)
Controls (*n* = 5)	303.8 ± 9.46	339.5 ± 14.97	5.53 ± 0.24	5.63 ± 0.27
3-day diabetics (*n* = 5)	273.0 ± 11.15	274.3 ± 8.09	6.17 ± 0.37	23.03 ± 2.63^∗∗∗∗^
5-day diabetics (*n* = 5)	293 ± 13.7	301.7 ± 11.63	5.87 ± 0.29	23.14 ± 2.01^∗∗∗∗^
10-day diabetics (*n* = 5)	310.5 ± 4.27	346 ± 9.06	4.9 ± 0.29	23.95 ± 1.18^∗∗∗∗^

^∗∗∗∗^ *P* < 0.0001 (initial versus final).

**Table 2:** Weight and glycaemic characteristics of the animal groups of the chronic diabetic experiment.

	Body weight (g) ± SEM		Blood glucose concentration (mM) ± SEM	
	Initial	Final	Initial	Final (average)
Controls (*n* = 5)	232.2 ± 7.29	486 ± 4.93^∗∗∗∗^	7.08 ± 0.22	6.3 ± 0.13
STZ-treated diabetics (*n* = 6)	235.3 ± 10.48	382.7 ± 3.53^∗∗∗∗^^oooo^	6.6 ± 0.1	23.31 ± 0.53^∗∗∗∗^^oooo^

^∗∗∗∗^ *P* < 0.0001 (initial versus final), ^oooo^*P* < 0.0001 (between final values).

## Abbreviations

ELISA: Enzyme-linked immunosorbent assays
GI: Gastrointestinal
HO: Heme oxygenase
IR: Immunoreactive
nNOS: Neuronal nitric oxide synthase
PB: Phosphate buffer
ROS: Reactive oxygen species
STZ: Streptozotocin.
